# GNG12 regulates PD‐L1 expression by activating NF‐κB signaling in pancreatic ductal adenocarcinoma

**DOI:** 10.1002/2211-5463.12784

**Published:** 2020-01-21

**Authors:** Juan Li, Can Jin, Chuanxin Zou, Xu Qiao, Peng Ma, Di Hu, Wenqin Li, Jun Jin, Xin Jin, Ping Fan

**Affiliations:** ^1^ Department of General Medicine The Second Clinical Medical College Jingzhou Central Hospital Yangtze University Jingzhou China; ^2^ Department of Gastroenterology The Second Clinical Medical College Jingzhou Central Hospital Yangtze University Jingzhou China; ^3^ Digestive Endoscopy Center The Second Clinical Medical College Jingzhou Central Hospital Yangtze University Jingzhou China; ^4^ Department of Oncology No. 99 Hospital of Joint Logistics Support Force of PLA Zhangzhou China; ^5^ Cancer Center Tongji Medical College Union Hospital Huazhong University of Science and Technology Wuhan China

**Keywords:** GNG12, p65, pancreatic ductal adenocarcinoma, PD‐L1

## Abstract

Pancreatic ductal adenocarcinoma (PDAC) is one of the most aggressive solid tumors in the digestive system. A greater understanding of the pathogenesis of PDAC may facilitate the search for new therapeutic targets. Guanine nucleotide‐binding protein subunit gamma‐12 (GNG12) belongs to the G protein family and participates in the modulation of the inflammatory signaling cascade. However, the cancer‐related function and clinical relevance of GNG12 in PDAC have not previously been reported. Here, we investigated the clinical significance of GNG12 in PDAC using the Oncomine web tool, the gene expression profiling interactive analysis tool and tissue microarray (TMA). GNG12 expression was observed to be higher in PDAC patient specimens than in nontumor pancreatic tissues, and high expression of GNG12 was associated with poor prognosis. We subsequently show that GNG12 promotes pancreatic cancer cell growth *in vivo* and *in vitro*, as evaluated using 3‐(4,5‐dimethylthiazol‐2‐yl)‐5‐(3‐carboxymethoxyphenyl)‐2‐(4‐sulfophenyl)‐2H‐tetrazolium, inner salt assays, colony formation assays and a xenograft mouse model. Furthermore, our results suggest that GNG12 activates nuclear factor‐κB signaling and modulates the immune response. Collectively, our findings suggest that GNG12 may be suitable as a new prognosis‐related biomarker and a promising target for treatment of pancreatic cancer.

## Graphical abstract

Pancreatic ductal adenocarcinoma is one of the most aggressive solid tumors in the digestive system. Guanine nucleotide‐binding protein subunit gamma‐12 (GNG12) expression was observed to be higher in pancreatic ductal adenocarcinoma patient specimens than in nontumor pancreatic tissues, and high expression of GNG12 was associated with poor prognosis. Furthermore, our results suggest that GNG12 activates nuclear factor‐κB signaling and modulates the immune response.

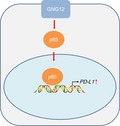

**Abbreviations**
GEPIA: gene expression profiling interactive analysis
GNG12: guanine nucleotide‐binding protein subunit gamma‐12
IHC: immunohistochemistry
MTS: 3‐(4,5‐dimethylthiazol‐2‐yl)‐5‐(3‐carboxymethoxyphenyl)‐2‐(4‐sulfophenyl)‐2H‐tetrazolium, inner salt
NF‐κB: nuclear factor‐κB
PDAC: pancreatic ductal adenocarcinoma
PD‐L1: programmed death‐ligand 1
shRNA: short hairpin RNA
TMA: tissue microarray

Pancreatic ductal adenocarcinoma (PDAC) is the most lethal solid tumor in the digestive system 1, 2. Due to rapid metastasis after surgical resection and chemotherapy resistance, the occurrence rate of PDAC is almost equal to its death‐causing degree, and this situation has not changed over the last couple decades 1, 2, 3. Therefore, exploring the pathogenesis of PDAC could help find new therapeutic targets for pancreatic cancer that help improve the treatment effect on patients with cancer.

Guanine nucleotide‐binding protein subunit gamma‐12 (GNG12) is one of the G protein family members and acts as a modulator in a variety of transmembrane signal pathways 4. Moreover, there is evidence that the protein kinase C–dependent phosphorylation of GNG12 regulates the interaction between Ca21 and cyclic AMP, which means that GNG12 has a novel regulatory function in cells 4. Furthermore, research has demonstrated that GNG12 can block the inflammatory response induced by lipopolysaccharide 5. Also, the aberrant expression of GNG12 has recently been shown to activate the mammalian target of rapamycin signaling cascade to promote cell proliferation and casein synthesis 6.

The cancer‐related function and clinical relevance of GNG12 in PDAC have remained unknown so far, but identifying them would be of massive significance toward finding a new treatment. In this study, we showed that GNG12 increased in pancreatic cancer patient specimens compared with the nontumor pancreatic tissues. GNG12 promoted pancreatic cancer cell growth *in vivo* and *in vitro*. In addition, we found that GNG12 activated the nuclear factor‐κB (NF‐κB) signaling pathway and increased the programmed death‐ligand 1 (PD‐L1) expression level in pancreatic cancer cells. Our findings show that overexpressed GNG12 promotes cancer cell proliferation and activates the NF‐κB signaling pathway and immune response in pancreatic cancer.

## Materials and methods

### Cells and cell culture

Human PDAC cell lines PANC‐1 (catalog no. SCSP‐535; Chinese Academy of Science Cell Bank, Shanghai, China) and BxPC‐3 (catalog no. TCHu 12; Chinese Academy of Science Cell Bank) were maintained in Dulbecco’s modified Eagle’s medium (Thermo Fisher Scientific, Shanghai, China) containing 10% FBS (Thermo Fisher Scientific). All pancreatic cancer cell lines were cultured in an incubator supplemented with 5% CO_2_ at 37 °C.

### Western blot analysis

Human pancreatic cancer cells (PANC‐1 and BxPC‐3) were washed with 1× PBS, harvested from culture dishes and lysed with 1× radioimmunoprecipitation assay (RIPA) buffer (Cell Signaling Technology, Danvers, MA, USA) for 15 min on ice. The supernatant obtained after centrifugation at 12 000 ***g*** was sent for quantification with a bicinchoninic acid Protein Assay Kit (catalog no. PA115; Tiangen Biotech, Beijing, China) according to the manufacturer’s protocol. Eighty micrograms of protein was separated using 8–10% SDS/PAGE gel and then transferred to a poly(vinylidene difluoride) membrane, where it was washed with 1× TBST, blocked with 5% nonfat milk at room temperature for 1 h and incubated with corresponding primary antibodies at 4 °C for more than 10 h. A second incubation was next performed with a secondary antibody at room temperature for 1 h, followed by visualization of target protein bands using a Chemiluminescent Western Blot Detection Kit (cat no. 32209; Thermo Fisher Scientific, Waltham, MA, USA).

### Real‐time RT‐PCR

The human pancreatic cancer cells (PANC‐1 and BxPC‐3) were washed with 1× PBS and immersed with 1 mL TRIzol reagent (Thermo Fisher Scientific, USA) for 20 min on ice. The RNA in the TRIzol reagent was sent for reverse transcription to generate the cDNA by using PrimeScript™ RT reagent Kit (cat no. RR037A; Shigo, Japan). Then the real‐time RT‐PCR was performed by adding the cDNA to the TB Green™ Fast qPCR Mix kit (cat no. RR430A; Shigo, Japan). The −∆∆Ct method was used for quantification, and β‐actin was used for the housekeeping gene. The primers for RT‐PCR are provided in Table 1.

**Table 1:** Sequences of quantitative RT‐PCR primers.

Species	Gene	Forward (5′–3′)	Reverse (5′–3′)
Human	*β‐actin*	CCCTGGCTCCTAGCACCAT	AGAGCCACCAATCCACACAGA
Human	*GNG12*	GCAAAACAGCAAGCACCAAC	CTATCAGCAAAGGGTCACTCC
Human	*PD‐L1*	CTGAACGCCCCATACAACAA	CTTGGAATTGGTGGTGGTGG
Human	*TNF*	GTCAACCTCCTCTCTGCCAT	CCAAAGTAGACCTGCCCAGA
Human	*IL‐1a*	TGATCAGTACCTCACGGCTG	TGGTCTTCATCTTGGGCAGT
Human	*IL‐6*	AGTCCTGATCCAGTTCCTGC	CTACATTTGCCGAAGAGCCC
Human	*CD83*	TGCTCCGAAGATGTGGACTT	AGAGTGCACCTGTATGTCCC
Human	*GADD45B*	CAGAAGATGCAGACGGTGAC	AAGGACTGGATGAGCGTGAA
Human	*BCL2L1*	AAGAGAACAGGACTGAGGCC	TTGCTTTACTGCTGCCATGG
Human	*CXCL5*	GTGTTGAGAGAGCTGCGTTG	AGGGGCTTCTGGATCAAGAC
Human	*CXCR1*	ATCTGTCCCTGCCCTTCTTC	GACGACAGCAAAGATGACCC
Human	*FOS*	GCTTCAACGCAGACTACGAG	AGTGACCGTGGGAATGAAGT
Human	*NR4A2*	CACTTCTCTCCCCAGCTTCA	CGGCATCATCTCCTCAGACT

### Antibodies, chemicals and plasmids

The p65 antibody (8242) (1 : 1000 for western blotting) and AKT antibody (9272) (1 : 2000 for western blotting) were obtained from Cell Signaling Technology; PD‐L1 antibody (ab205921) (1 : 1000 for western blotting), GNG12 antibody (ab154698) (1 : 1000 for western blotting) and GAPDH antibody (ab8245) (1 : 5000 for western blotting) were acquired from Abcam (Cambridge, MA, USA). A pcDNA3.1 backbone was mixed with the cDNA of GNG12 to generate the Flag‐GNG12 vector.

### RNA interference

The lentivirus vector–based short hairpin RNAs (shRNAs) (Sigma‐Aldrich, St. Louis, MO, USA) were transfected to 293T cells. After 72 h of transfection, the media of 239T cells containing virus were harvested. For infection of the pancreatic cancer cells, the culture medium of pancreatic cancer cells was replaced with the above viral medium. After 72‐h infection and puromycin selection, the pancreatic cancer cells were harvested for other experiments. The information for the shRNA sequence is provided in Table 2.

**Table 2:** Sequences of gene‐specific shRNAs.

shGNG12#1	5 ′ ‐CCGGCCGATATGTCAGGACCTAAATCTCGAGATTTAGGTCCTGACATATCGGTTTTTTG‐3 ′
shGNG12#2	5 ′ ‐CCGGGCTCATGTTCTCTACTGGATTCTCGAGAATCCAGTAGAGAACATGAGCTTTTTTG‐3 ′
shp65	5 ′ ‐CCGGGCTCATGTTCTCTACTGGATTCTCGAGAATCCAGTAGAGAACATGAGCTTTTTTG‐3 ′

### Cell proliferation assay

The pancreatic cancer cell growth rate was analyzed by using the 3‐(4,5‐dimethylthiazol‐2‐yl)‐5‐(3‐carboxymethoxyphenyl)‐2‐(4‐sulfophenyl)‐2H‐tetrazolium, inner salt (MTS) assay (Abcam). In brief, pancreatic cancer cells (0.5 × 10^4^ cells per well) were plated in 96‐well plates. After 24 h, MTS reagent (Abcam) was added to each well and incubated at 37 °C for 1 h. The microplate reader was used to measure the *A*_490 nm_.

### Xenograft mouse model

The 4‐ to 5‐week‐old nude mice (Vitalriver, China) were randomly divided into shControl or shGNG12 group (*n* = 6 per group). The pancreatic cancer cells (1 × 10^7^) infected with control or GNG12 shRNAs were subcutaneously injected in the left dorsal flank of the mice. The tumor volume [length × (width^2^)/2] was measured every other day for 21 days. The xenografts mouse protocol was approved by the Institutional Animal Care and Use Committee of Tongji Medical College, Huazhong University of Science and Technology.

### Immunohistochemistry

The TMA slides were purchased from Outdo Biobank (HPan‐Ade060CD‐01; Shanghai, China). The TMA specimens were immunostained with GNG12 antibodies (ab154698, dilution 1 : 150; Abcam) as described previously 7. Staining intensity was scored in a blinded fashion: 1 = weak staining at ×100 magnification but little or no staining at ×40 magnification; 2 = medium staining at ×40 magnification; and 3 = strong staining at ×40 magnification 7. The degree of immunostaining was reviewed and scored by two independent pathologists who were blinded to the clinical details. The scores were determined by the percentage of positive cells multiplied by the staining intensity.

### Statistical analysis

All grouped data are presented as the means ± standard deviation (SD). Comparisons between groups were determined with one‐way or two‐way ANOVA using graphpad prism 5 software (GraphPad Software, San Diego, CA, USA). *P* < 0.05 was considered statistically significant.

## Results

### Overexpressed GNG12 is associated with poor prognosis in PDAC

**Figure 1:**
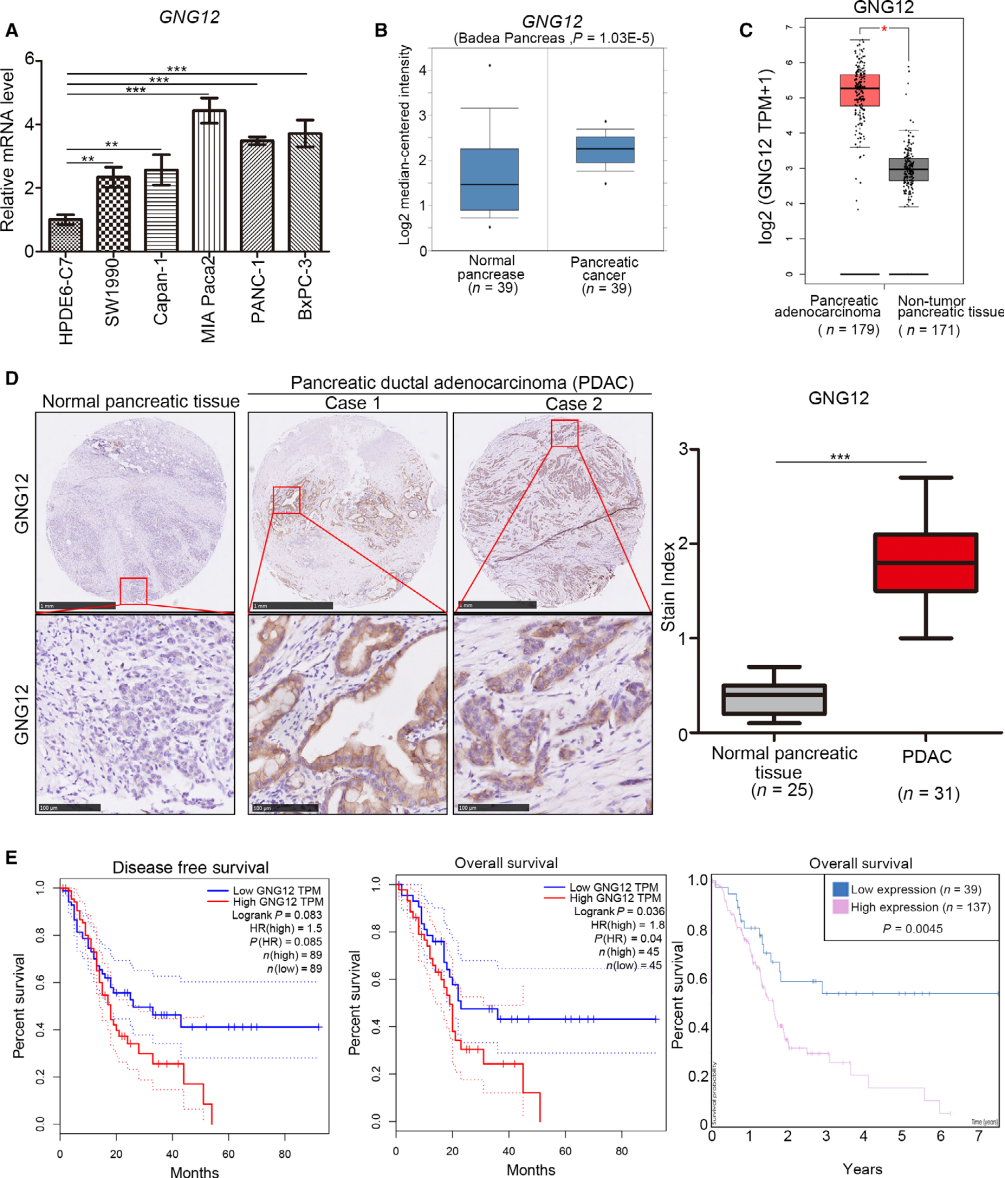
Overexpressed GNG12 is associated with poor prognosis in PDAC. (A) The normal pancreatic cell lines and pancreatic cancer cell lines were collected for quantitative RT‐PCR analysis. Data presented are the mean values ± SD (*n* = 3). Analysis performed using one‐way ANOVA with Tukey’s multiple comparisons *post hoc* test. **P < 0.01; ***P < 0.001. (B) The mRNA expression level of GNG12 was measured by the Oncomine web tool. *P* values are as indicated. (C) The mRNA expression level of GNG12 was measured by the GEPIA web tool. Asterisk indicates significant. (D) The IHC image and stain index of GNG12 by using TMA sections. Scale bars: 1 mm (upper panels); 100 µm (lower panels). *P* values are shown as indicated. Analysis was performed using the Shapiro–Wilk normality test. ***P < 0.001. (E) The disease‐free and overall survival of patients with pancreatic cancer were analyzed using the GEPIA web tool and the Human Protein Atlas. *P* values are shown as indicated. Disease‐free survival rate and overall survival rate were analyzed using log rank test and Mantel–Cox test.

Because GNG12 in cancer is poorly understood, especially in pancreatic cancer, we first evaluated the mRNA expression of GNG12 in pancreatic cancer cell lines and normal human pancreatic duct epithelial cells. Our data show that there were higher mRNA expression levels of GNG12 in pancreatic cancer cell lines, especially in MIA Paca‐2, PANC‐1 and BxPC‐3 cells, than in HPDE6‐C7 cells (normal human pancreatic duct epithelial cells) (Fig. 1A).

Next, we compared the GNG12 mRNA levels in PDAC with those in nontumor pancreatic tissues using the Oncomine and gene expression profiling interactive analysis (GEPIA) web tool 8. Our data revealed that GNG12 expression in PDAC was much higher than in nontumor pancreatic tissues (Fig. 1B,C).

Meanwhile, the protein levels of GNG12 were detected by immunohistochemistry (IHC) from a PDAC TMA [nontumor pancreatic tissues (*n* = 25) and PDAC specimens (*n* = 31)] (cat no. XT14‐029; Outdo Biobank). IHC results indicated that GNG12 has higher protein levels in PDAC specimens than in nontumor pancreatic tissues (Fig. 1D).

We then investigated the disease‐free survival and overall survival rates of patients with PDAC with different GNG12 expressions using the Human Protein Atlas and the GEPIA web tool. Our data revealed that the GNG12 high‐expression group had a shorter survival time than the GNG12 low‐expression group (Fig. 1E). Therefore, our data suggest that GNG12 increases in PDAC, and overexpressed GNG12 correlates with unfavorable prognosis in pancreatic cancer.

### Abnormally overexpressed GNG12 promotes pancreatic cancer cell growth

**Figure 2:**
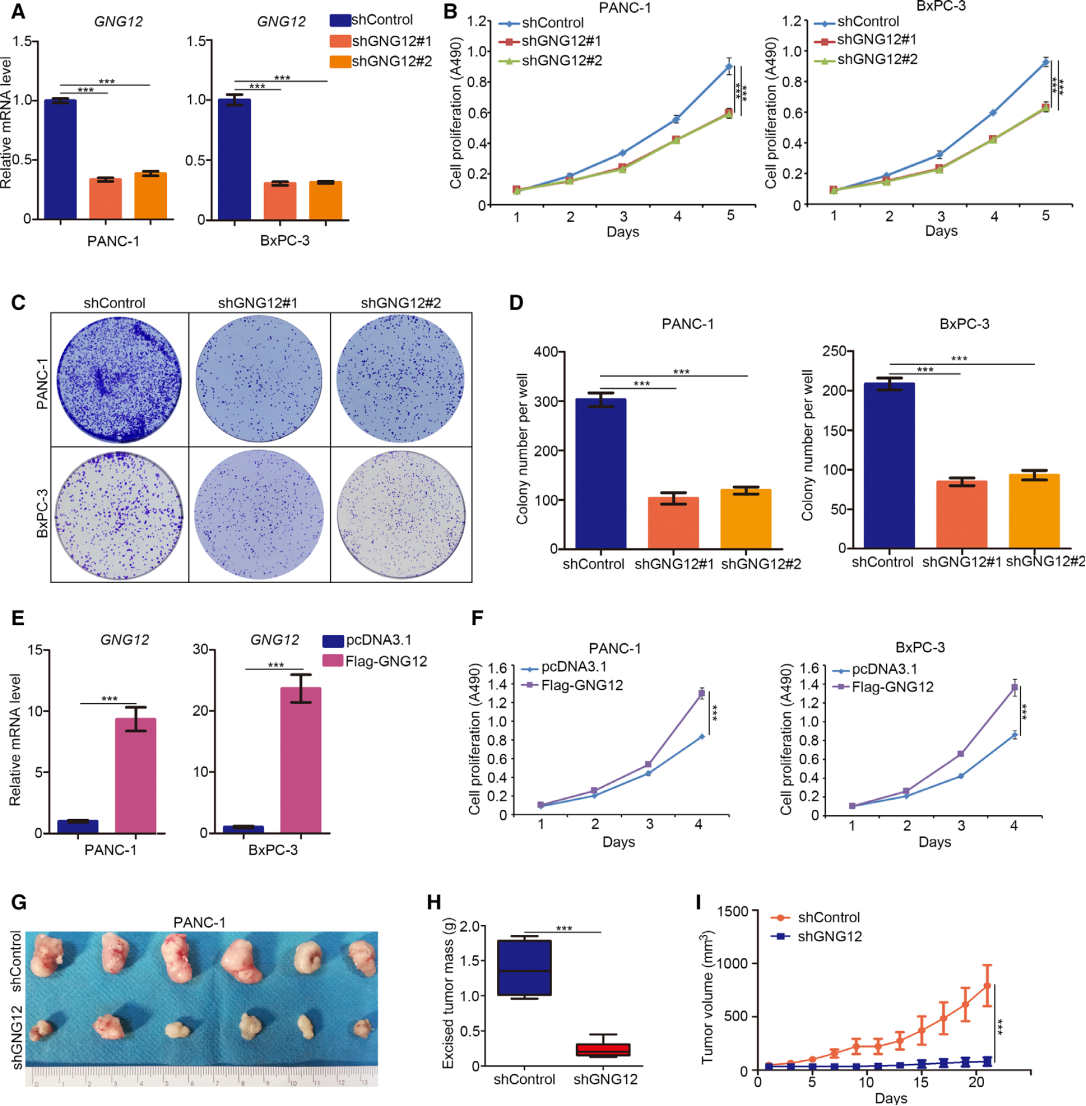
Abnormally overexpressed GNG12 promotes pancreatic cancer cell growth. (A–D) BxPC‐3 and PANC‐1 cells were transfected with indicated constructs. Seventy‐two hours after infection and puromycin selection, cells were subjected to quantitative RT‐PCR analysis (A), MTS assay (B) and colony formation assay (C, D). Data presented are the mean values ± SD (*n* = 3). Analysis was performed using one‐way ANOVA with Tukey’s multiple comparisons *post hoc* test. ***P < 0.001. (E, F) BxPC‐3 and PANC‐1 cells were transfected with indicated plasmids. Twenty‐four hours posttransfection, cells were collected for quantitative RT‐PCR analysis (E) and MTS assay (F). The data shown are the mean values ± SD (*n* = 3). Analysis was performed using one‐way ANOVA with Tukey’s multiple comparisons *post hoc* test. ***P < 0.001. (G–I) PANC‐1 cells were transfected with shControl or GNG12‐specific shRNA. Seventy‐two hours after transfection and puromycin selection, cells were subcutaneously injected into nude mice for xenografts assay. After 21 days, the tumors were harvested, photographed (G) and measured (H, I). The data are presented as the mean ± SD (*n* = 6). Analysis was performed using one‐way ANOVA with Tukey’s multiple comparisons *post hoc* test. ***P < 0.001.

Having identified that overexpressed GNG12 correlated with the poor prognosis of PDAC, we needed to establish the tumorigenesis‐related effect of GNG12 in PDAC. First, we knocked down GNG12 with shRNAs in pancreatic cancer cells and subjected the cells to the MTS and colony formation assays (Fig. 2A). Our data showed that knocking down GNG12 significantly inhibited pancreatic cancer cell growth *in vitro* (Fig. 2B–D). In contrast, overexpressed GNG12 induced by GNG12 plasmid transfection up‐regulated PANC‐1 and BxPC‐3 cell growth rates (Fig. 2E,F).

Furthermore, PANC‐1 cells with or without knocked‐down GNG12 were transfected into nude mice subcutaneously for the xenografts assay, to verify the tumor growth‐promoting effect of GNG12. Our results suggest that the down‐regulation of GNG12 impeded xenograft growth *in vivo* (Fig. 2G–I). These findings indicate that GNG12 could promote pancreatic cancer cell growth *in vivo* and *in vitro*.

### GNG12 activates the NF‐κB pathway in pancreatic cancer cells

**Figure 3:**
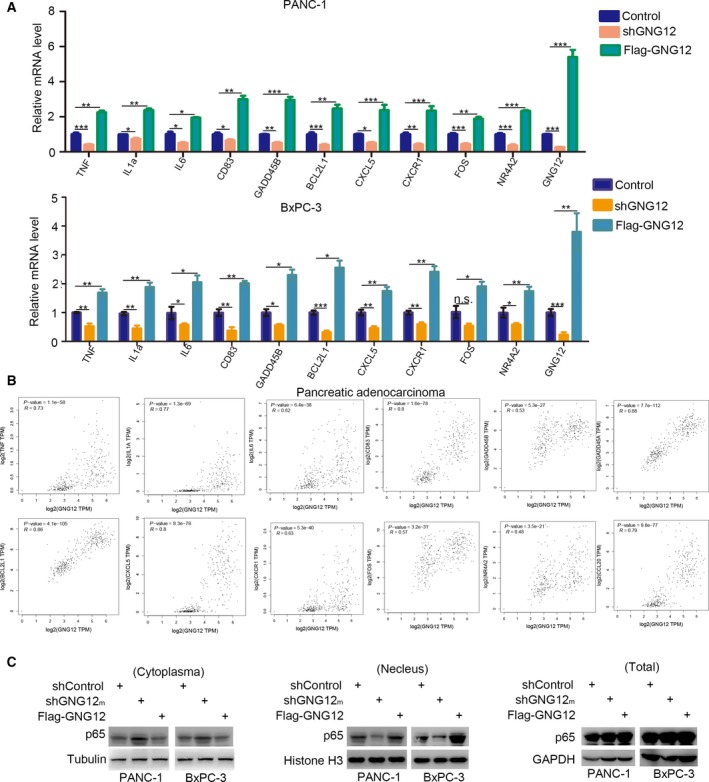
GNG12 activates the NF‐κB pathway in pancreatic cancer cells. (A) PANC‐1 and BxPC‐3 cells were transfected with indicated constructs. Seventy‐two hours after transfection and puromycin selection, cells were harvested for quantitative RT‐PCR analysis. Data presented are the mean values ± SD (*n* = 3). Analysis was performed using two‐way ANOVA with Bonferroni *post hoc* test. *P < 0.05; **P < 0.01; ***P < 0.001. (B) The correlation between GNG12 and NF‐κB pathway‐related genes was analyzed by the GEPIA web tools. *P* value is as indicated. (C) PANC‐1 and BxPC‐3 cells were transfected with indicated constructs. Seventy‐two hours after transfection and puromycin selection, cells were harvested for western blotting analysis. n.s., not significant.

The dysregulation of NF‐κB signaling is one of the major characteristics of pancreatic cancers 9. Activated NF‐κB pathways have been reported to contribute to regulating inflammatory responses and promoting pancreatic cancer proliferation 10. Given that GNG12 is involved in modulating inflammatory responses, we further explored the underlying mechanism of GNG12‐induced pancreatic cancer cell proliferation. We first examined whether GNG12 regulated NF‐κB–related genes in pancreatic cancer cells. Our results revealed that knocking down GNG12 decreased the number of NF‐κB pathway‐related gene expressions (including tumor necrosis factor [TNF], interleukin‐1a [IL‐1a], IL‐6, CD83, GADD45B, BCL2L1, CXCL5, CXCR1, FOS and NR4A2) 10, 11 in both PANC‐1 and BxPC‐3 cells (Fig. 3A). In contrast, overexpressed GNG12 up‐regulated these genes’ expression in pancreatic cancer cell lines (Fig. 3A). Besides, we found that there were positive correlations between these genes and GNG12 levels in pancreatic cancer specimens (Fig. 3B). Furthermore, our data indicated that the repression of GNG12 resulted in decreased nuclear portions of p65 in PANC‐1 and BxPC‐3 cells, but the overexpression of GNG12 led to increased p65 in the nucleus (Fig. 3C), which might be an indicator of how GNG12 activates NF‐κB signaling in pancreatic cancer.

### GNG12 promotes PD‐L1 expression in pancreatic cancer through the NF‐κB pathway

**Figure 4:**
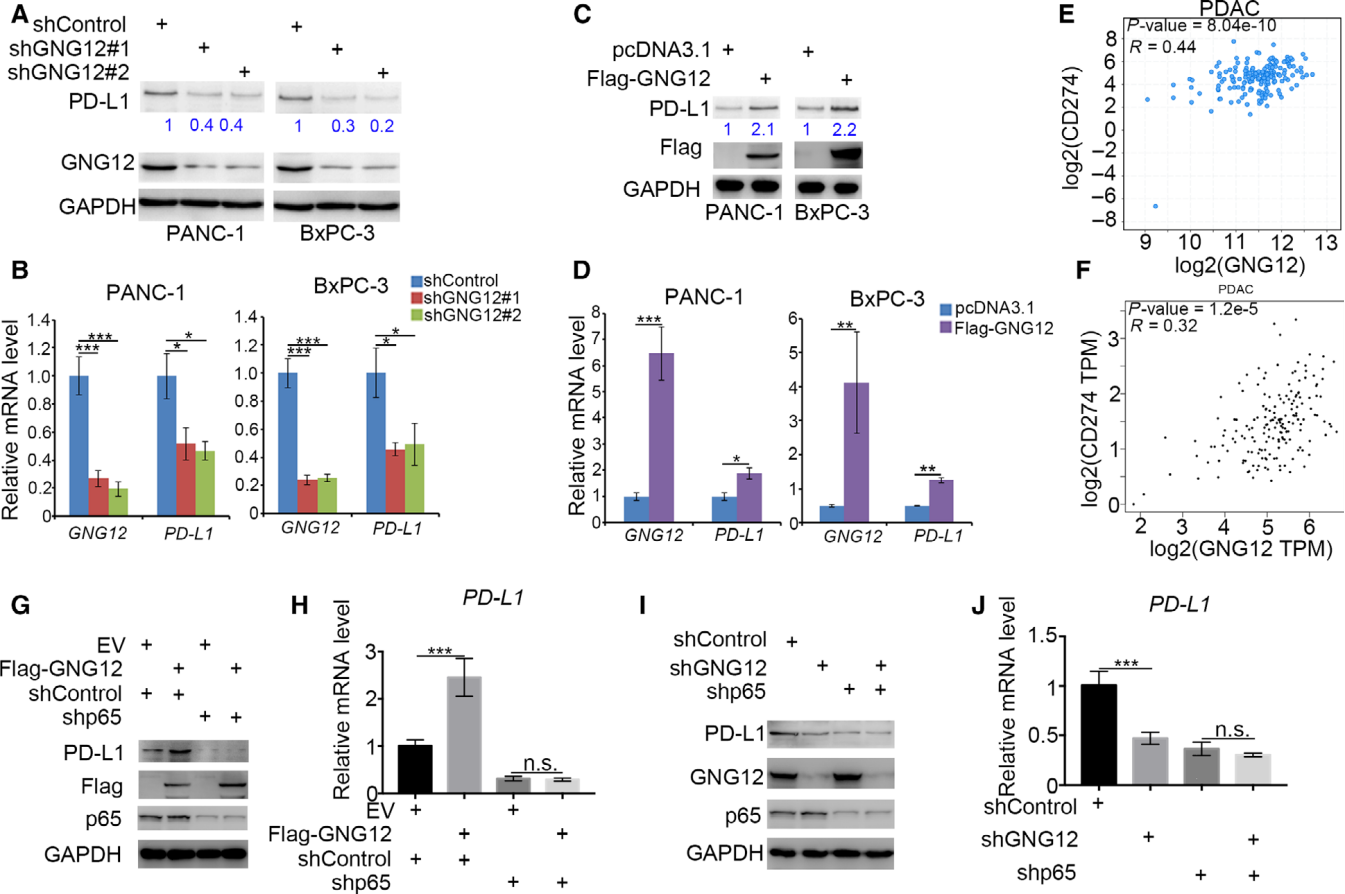
GNG12 promotes PD‐L1 expression in pancreatic cancer through the NF‐κB pathway. (A, B) PANC‐1 and BxPC‐3 cells were transfected with indicated constructs. Seventy‐two hours after transfection and puromycin selection, cells were harvested for quantitative RT‐PCR analysis (A) and western blotting analysis (B). The data shown are the mean values ± SD from three replicates. Analysis was performed using one‐way ANOVA with Tukey’s multiple comparisons *post hoc* test. *P < 0.05; **P < 0.01; ***P < 0.001. (C, D) PANC‐1 and BxPC‐3 cells were transfected with indicated constructs. Seventy‐two hours after transfection and puromycin selection, cells were harvested for quantitative RT‐PCR analysis (C) and western blotting analysis (D). The data shown are the mean values ± SD from three replicates. Analysis was performed using one‐way ANOVA with Tukey’s multiple comparisons *post hoc* test. **P < 0.01; ***P < 0.001. (E and F) The Cancer Genome Atlas datasets (E) and the GEPIA web tool (F) were used to determine the correlation between the mRNA expression levels of PD‐L1 and GNG12 in human pancreatic cancer samples. (G, H) PANC‐1 and BxPC‐3 cells were transfected with indicated constructs. Seventy‐two hours after transfection and puromycin selection, cells were harvested for quantitative RT‐PCR analysis (G) and western blotting analysis (H). The data shown are the mean values ± SD from three replicates. Analysis was performed using one‐way ANOVA with Tukey’s multiple comparisons *post hoc* test. ***P < 0.001. (I, J) PANC‐1 and BxPC‐3 cells were transfected with indicated constructs. Seventy‐two hours after transfection and puromycin selection, cells were harvested for quantitative RT‐PCR analysis (I) and western blotting analysis (J). The data shown are the mean values ± SD from three replicates. Analysis was performed using one‐way ANOVA with Tukey’s multiple comparisons *post hoc* test. ***P < 0.001. n.s., not significant.

Previous studies have reported that the activation of NF‐κB signaling increases PD‐L1 expression. Given that GNG12 activates the NF‐κB signaling pathway, we sought to know whether GNG12 modulates PD‐L1. Intriguingly, knocking down GNG12 down‐regulated the protein and mRNA levels of PD‐L1 transcriptionally in pancreatic cancer cells (Fig. 4A,B). Conversely, GNG12 overexpression increased PD‐L1 expression (Fig. 4C,D).

Furthermore, we analyzed the correlation of the mRNA levels between GNG12 and PD‐L1 (also known as CD274) using The Cancer Genome Atlas datasets (Fig. 4E) and GEPIA web tool (Fig. 4F). Our findings showed that GNG12 correlated positively with PD‐L1 in PDAC specimens (Fig. 4E,F). In addition, we revealed that the up‐regulation of PD‐L1 induced by GNG12 overexpression or the down‐regulation of PD‐L1 caused by knocking down GNG12 could both be diminished by knocking down p65 in PANC‐1 cells (Fig. 4G–J). These data suggest that GNG12 promotes PD‐L1 expression via the NF‐κB pathway in pancreatic cancer.

## Discussion

The NF‐κB family of the transcription factor consists of five members, including p65, p50/p105, p52/p100, c‐Rel and RelB 12. The abnormal activation of the NF‐κB pathway contributes to the dysregulation of cancer cell metabolism, cell cycle, apoptosis and chemoresistance 13. Reports suggest that the canonical NF‐κB signal is overactivated in 70% of pancreatic cancer cell lines, which results in cancer cell growth and lymphovascular and neural invasion 14, 15. Thus, exploring the underlying mechanism of how the NF‐κB signal is activated is critical to understanding the pathogenesis of pancreatic cancer. Our group previously showed that the G protein–coupled receptor activates the NF‐κB signal to regulate pancreatic cancer proliferation, angiogenesis and gemcitabine chemoresistance 9. In this study, we revealed that GNG12 acted as an activator of the NF‐κB pathway in pancreatic cancer cells, but the specific mechanism needs to be studied further.

Recently, immunotherapy, especially the use of immune checkpoint inhibitors, is a novel therapeutic strategy for cancer treatment and makes profound progress in prolonging the survival time of various types of tumor 16. A number of clinical trials are currently being conducting to explore the safety and efficacy of immune checkpoint inhibitors for gastrointestinal cancer 16. Single‐agent immune checkpoint inhibitions, including programmed death 1 antibody or PD‐L1 antibodies, show nonclinical benefits for patients with pancreatic cancer 17. It has been well documented that genes alteration associated with mesenchymal transition, angiogenesis or hypoxia was responsible for the resistance of immune checkpoint inhibitions 16. Importantly, overexpressed PD‐L1 is considered to be one of the primary reasons for the failure of immune checkpoint immunotherapy 18. The specific mechanism of PD‐L1 regulation in pancreatic cancer has not been elucidated fully. Existing reports show that tumor necrosis factor‐α, secreted by macrophages, increases PD‐L1 expression in pancreatic cancer 19. Previously, we found that the RB–p65 axis 10, FUBP1–Myc axis 20, HAT1–BRD4 axis 21 or HDAC3 22 regulated PD‐L1 expression in pancreatic cancer. In this study, we demonstrated that GNG12 increased PD‐L1 transcriptionally and modulated the immune response to pancreatic cancer.

In summary, we studied the biological role of GNG12 in pancreatic cancer. Our data indicate that GNG12 increased in PDAC specimens, and overexpressed GNG12 was associated with poor prognosis of pancreatic cancer. We also showed that GNG12 promoted PDAC tumor growth *in vivo* and *in vitro*. In addition, we found that GNG12 played a crucial role in activating NF‐κB singling and modulating the immune response. Therefore, we suggest that GNG12 might be a new prognosis‐related biomarker and a promising candidate for pancreatic cancer treatment.

## Conflict of interest

The authors declare no conflict of interest.

## Author contributions

JL, CJ, CZ, XQ and PM performed the experiments. DH, WL and JJ collected and analyzed the data. XJ and PF conceived the study and wrote the paper.

## References

[1] Siegel RL , Miller KD and Jemal A (2017) Cancer statistics, 2017. CA Cancer J Clin 67, 7–30. 28055103 10.3322/caac.21387

[2] Bray F , Ferlay J , Soerjomataram I , Siegel RL , Torre LA and Jemal A (2018) Global cancer statistics 2018: GLOBOCAN estimates of incidence and mortality worldwide for 36 cancers in 185 countries. CA Cancer J Clin 68, 394–424. 30207593 10.3322/caac.21492

[3] Silvestris N , Gnoni A , Brunetti AE , Vincenti L , Santini D , Tonini G , Merchionne F , Maiello E , Lorusso V , Nardulli P *et al* (2014) Target therapies in pancreatic carcinoma. Curr Med Chem 21, 948–965. 23992319 10.2174/09298673113209990238

[4] Yasuda H , Lindorfer MA , Myung CS and Garrison JC (1998) Phosphorylation of the G protein gamma12 subunit regulates effector specificity. J Biol Chem 273, 21958–21965. 9705336 10.1074/jbc.273.34.21958

[5] Larson KC , Lipko M , Dabrowski M and Draper MP (2010) Gng12 is a novel negative regulator of LPS‐induced inflammation in the microglial cell line BV‐2. Inflamm Res 59, 15–22. 19568691 10.1007/s00011-009-0062-2

[6] Luo C , Zhao S , Dai W , Zheng N and Wang J (2018) Proteomic analyses reveal GNG12 regulates cell growth and casein synthesis by activating the Leu‐mediated mTORC1 signaling pathway. Biochim Biophys Acta Proteins Proteom 1866, 1092–1101. 30282607 10.1016/j.bbapap.2018.08.013

[7] Jin X , Pan Y , Wang L , Zhang L , Ravichandran R , Potts PR , Jiang J , Wu H and Huang H (2017) MAGE‐TRIM28 complex promotes the Warburg effect and hepatocellular carcinoma progression by targeting FBP1 for degradation. Oncogenesis 6, e312. 28394358 10.1038/oncsis.2017.21 PMC5520498

[8] Tang Z , Li C , Kang B , Gao G , Li C and Zhang Z (2017) GEPIA: a web server for cancer and normal gene expression profiling and interactive analyses. Nucleic Acids Res 45, W98–W102. 28407145 10.1093/nar/gkx247 PMC5570223

[9] Wang L , Zhou W , Zhong Y , Huo Y , Fan P , Zhan S , Xiao J , Jin X , Gou S , Yin T *et al* (2017) Overexpression of G protein‐coupled receptor GPR87 promotes pancreatic cancer aggressiveness and activates NF‐kappaB signaling pathway. Mol Cancer 16, 61. 28288630 10.1186/s12943-017-0627-6 PMC5348802

[10] Jin X , Ding D , Yan Y , Li H , Wang B , Ma L , Ye Z , Ma T , Wu Q , Rodrigues DN *et al* (2019) Phosphorylated RB promotes cancer immunity by inhibiting NF‐kappaB activation and PD‐L1 expression. Mol Cell 73, 22–35. e6. 30527665 10.1016/j.molcel.2018.10.034 PMC8968458

[11] Zhao D , Lu X , Wang G , Lan Z , Liao W , Li J , Liang X , Chen JR , Shah S , Shang X *et al* (2017) Synthetic essentiality of chromatin remodelling factor CHD1 in PTEN‐deficient cancer. Nature 542, 484–488. 28166537 10.1038/nature21357 PMC5448706

[12] Hoesel B and Schmid JA (2013) The complexity of NF‐kappaB signaling in inflammation and cancer. Mol Cancer 12, 86. 23915189 10.1186/1476-4598-12-86 PMC3750319

[13] Li Q , Yang G , Feng M , Zheng S , Cao Z , Qiu J , You L , Zheng L , Hu Y , Zhang T *et al* (2018) NF‐kappaB in pancreatic cancer: Its key role in chemoresistance. Cancer Lett 421, 127–134. 29432846 10.1016/j.canlet.2018.02.011

[14] Yuan P , He XH , Rong YF , Cao J , Li Y , Hu YP , Liu Y , Li D , Lou W and Liu MF (2017) KRAS/NF‐kappaB/YY1/miR‐489 signaling axis controls pancreatic cancer metastasis. Cancer Res 77, 100–111. 27793842 10.1158/0008-5472.CAN-16-1898

[15] Pramanik KC , Makena MR , Bhowmick K and Pandey MK (2018) Advancement of NF‐kappaB signaling pathway: a novel target in pancreatic cancer. Int J Mol Sci 19, 3890. 10.3390/ijms19123890 PMC6320793 30563089

[16] Basile D , Garattini SK , Bonotto M , Ongaro E , Casagrande M , Cattaneo M , Fanotto V , De Carlo E , Loupakis F , Urbano F *et al* (2017) Immunotherapy for colorectal cancer: where are we heading? Expert Opin Biol Ther 17, 709–721. 28375039 10.1080/14712598.2017.1315405

[17] Feng M , Xiong G , Cao Z , Yang G , Zheng S , Song X , You L , Zheng L , Zhang T and Zhao Y (2017) PD‐1/PD‐L1 and immunotherapy for pancreatic cancer. Cancer Lett 407, 57–65. 28826722 10.1016/j.canlet.2017.08.006

[18] Lu C , Paschall AV , Shi H , Savage N , Waller JL , Sabbatini ME , Oberlies NH , Pearce C and Liu K (2017) The MLL1‐H3K4me3 axis‐mediated PD‐L1 expression and pancreatic cancer immune evasion. J Natl Cancer Inst 109. 10.1093/jnci/djw283 PMC5291187 28131992

[19] Tsukamoto M , Imai K , Ishimoto T , Komohara Y , Yamashita YI , Nakagawa S , Umezaki N , Yamao T , Kitano Y , Miyata T *et al* (2019) PD‐L1 expression enhancement by infiltrating macrophage‐derived tumor necrosis factor‐alpha leads to poor pancreatic cancer prognosis. Cancer Sci 110, 310–320. 30426611 10.1111/cas.13874 PMC6317925

[20] Fan P , Ma J and Jin X (2018) Far upstream element‐binding protein 1 is up‐regulated in pancreatic cancer and modulates immune response by increasing programmed death ligand 1. Biochem Biophys Res Commun 505, 830–836. 30301530 10.1016/j.bbrc.2018.10.009

[21] Fan P , Zhao J , Meng Z , Wu H , Wang B , Wu H and Jin X (2019) Overexpressed histone acetyltransferase 1 regulates cancer immunity by increasing programmed death‐ligand 1 expression in pancreatic cancer. J Exp Clin Cancer Res 38, 47. 30709380 10.1186/s13046-019-1044-z PMC6359760

[22] Hu G , He N , Cai C , Cai F , Fan P , Zheng Z and Jin X (2019) HDAC3 modulates cancer immunity via increasing PD‐L1 expression in pancreatic cancer. Pancreatology 19, 383–389. 30670333 10.1016/j.pan.2019.01.011

